# Psycho-Social Factors in Patients with Cardiovascular Disease Attending a Family-Centred Prevention and Rehabilitation Programme: EUROACTION Model in Spain

**DOI:** 10.3390/life11020089

**Published:** 2021-01-26

**Authors:** Cristina Buigues, Ana Queralt, Jose Antonio De Velasco, Antonio Salvador-Sanz, Catriona Jennings, David Wood, Isabel Trapero

**Affiliations:** 1 Department of Nursing, University of Valencia, 46010 Valencia, Spain; ana.queralt@uv.es (A.Q.); isabel.trapero@uv.es (I.T.); 2 Frailty and Cognitive Impairment Group (FROG), University of Valencia, 46010 Valencia, Spain; 3 AFIPS Research Group, University of Valencia, 46022 Valencia, Spain; 4 Cardiology Service, Hospital General Universitario, 28222 Valencia, Spain; jdevelascor37@gmail.com; 5 Cardiology Department, Valencian Institute of Oncology Foundation, 46009 Valencia, Spain; asalvador@fivo.org; 6 National Institute for Prevention and Cardiovascular Health, National University of Ireland, H91 FF68 Galway, Ireland; catriona.jennings@nuigalway.ie (C.J.); d.wood2@imperial.ac.uk (D.W.); 7 Cardiovascular Medicine, National Heart and Lung Institute, Imperial College London (Hammersmith Campus), International Centre for Circulatory Health, London SW3 6LY, UK

**Keywords:** cardiovascular prevention, nurse-coordinated programme, illness perception, interdisciplinary, anxiety, depression

## Abstract

Background: Coronary heart disease (CHD) persists as the leading cause of death worldwide. Cardiovascular prevention and rehabilitation (CVPR) has an interdisciplinary focus, and includes not only in physiological components, but it also addresses psycho-social factors. Methods: The study analysed the Spanish psycho-social data collected during the EUROACTION study. In Spain, two hospitals were randomised in the Valencia community. Coronary patients were prospectively and consecutively identified in both hospitals. The intervention hospital carried out a 16-week CVPR programme, which aimed to assess illness perceptions and establish healthy behaviours in patients and their partners. Results: Illness perceptions were significantly and inversely associated with anxiety and depression. Low levels of anxiety were associated with better self-management of total cholesterol (*p* = 0.004) and low-density lipoprotein-cholesterol (*p* = 0.004). There was concordance at one year among patients and partners who participated in the programme related to anxiety (*p* < 0.001), fruit consumption (*p* < 0.001), and vegetable consumption (*p* < 0.001). Conclusions: The EUROACTION study emphasised the importance of assessing psycho-social factors in a CVPR programme and the inclusion of family as support in patients’ changes in behaviour.

## 1. Introduction

Despite numerous advances made in recent years, coronary heart disease (CHD) is still a serious health problem and remains the leading cause of death worldwide [1,2]. The risk for CHD includes both non-modifiable risk factors and modifiable risk factors, such as lifestyle and psycho-social factors. The prevalence of depression, anxiety, and psychological stress is estimated to be higher among patients with CHD, compared to the general adult population [3]. Moreover, depression and anxiety in patients with cardiac disease is associated with cardiac risk behaviour, such as smoking, obesity, excessive alcohol consumption, and medication non-adherence [4], and these cardiac risk factors are known to increase the risk of poor outcomes, e.g., morbidity or mortality in cardiac patients [5]. Consequently, lifestyle and psycho-social factors must be considered to be important in the evaluation of health care interventions.

A key message included in the latest European guidelines on cardiovascular disease prevention (2016) was that psychological interventions can counteract psycho-social stress and promote healthy behaviours and lifestyle. Further, the World Heart Federation estimates that with small changes in routine, such as healthier eating choices and a more balanced diet, exercising regularly, or giving up smoking, by 2025, premature deaths caused by cardiovascular disease (CVD) could be reduced by 25%. Therefore, it is critical to be aware of the psycho-social status of patients when they are discharged from the hospital, and the process they face in recovery from illness, in order to encourage these changes [6].

Cardiovascular prevention and rehabilitation (CVPR) has a robust evidence base for reducing cardiovascular mortality and morbidity, and it is essential for optimal CHD treatment. CVPR programmes significantly reduce depressive mood [7] and anxiety [8], as well as offer stress management in the context of comprehensive CVPR [9]. They are also important because CVPR improves the general perceived self-efficacy of patients who have suffered cardiac events, and consequently improves adherence to healthy behaviours that could decrease the risk of subsequent cardiac events [10]. The primary emphasis in CVPR should include modifying the health beliefs of patients, and motivating them to achieve and sustain behavioural changes [11]. Moreover, patients’ quality of life improves following participation in these programmes [12].

In addition, it is important to assess individuals’ understanding of their illness. Patients’ perceptions of their illness appear to play a pivotal role in choices regarding their own health. They have been found to be a predictor of depressive symptoms [13], and have been associated with quitting CVPR programmes, as well as the consequent levels of anxiety and depression [14]. Interventions to improve patients’ quality of life should focus on improving negative beliefs about CVPR and increasing understanding of the role of medication adherence to medication in preventing a future cardiac event. Patients’ beliefs at the start of CVPR are especially important for medication adherence. Physical and mental health at six months is negatively associated with baseline beliefs about CVPR [15].

Assessing illness perceptions is not only relevant in patients, but also in their partners. CVPR participants often report the need for assistance to make changes during the programme [16]. Clinical guidelines recommend involving partners or caregivers in the CVPR programme, if the patient agrees [17]. The importance of partners supporting the patient in the programme is often ignored; however, improving the perceptions of illness on the part of partners of heart attack patients may have important implications not only for patients’ recovery [5], but also for improving partners’ lifestyle habits [18]. For this reason, it is important to focus on analysing the influence of interpersonal behaviours of the partners [19].

High levels of anxiety in cardiac patients are often driven by maladaptive beliefs about their heart condition [20], and these misconceptions are frequently targeted during CVPR. Perceptions and misconceptions about illness by patients can determine the choice of coping strategies and affect adherence to treatment and the adoption of healthy behaviours [21,22,23].

The aim of the present study is to highlight the effects of an interdisciplinary, family-centred CVPR programme on modifying illness perceptions to achieve better outcomes and therefore improve the state of anxiety and depression of patients, as well as improving not only their lifestyle but also that of their partners.

## 2. Materials and Methods

Spain was one of the six participating countries in the European-cluster randomised, controlled trial called EUROACTION [24], which was conducted under the auspices of the European Society of Cardiology. In each of the six countries, two general hospitals were recruited. The pairs of hospitals in each country were comparable in relation to patients’ age, gender, and category of diagnostic distribution. They were randomised to either receive the intervention or to be monitored for their usual care provision. In Spain, Dr. Peset University Hospital in Valencia was selected as the intervention hospital, and the General Hospital of Alicante was selected as the usual care control group.

Consecutive patients were prospectively identified when they were admitted for an acute cardiac event or seen as an outpatient. Eligible patients (men and women) were less than 80 years of age and had CHD (i.e., acute coronary syndromes or exertional angina). Exclusion criteria for all patients were: severe heart failure, severe physical disability, and dementia. In the hospital intervention group, patients and their partners were recruited and invited to participate in a CVPR programme, which aimed at achieving a healthy lifestyle and control of risk factors set out in the European recommendations, for patients with CHD [25].

In the hospital control group, coronary patients were identified in the same way as in the intervention group, but only a randomly selected subsample performed an initial assessment. This was in order to reduce the influence of the programme on the study participants. The assessment was detailed, involving many questions about lifestyle habits which could stimulate a lot of discussion between the health professional and the patient. In the control group, patients did not receive any kind of additional support. Partners of patients in the control group were identified but not assessed at initial and 16-week assessments, for the same reason. They were assessed only at the one-year follow-up.

In the intervention hospital, the cardiologist and nurses recruited eligible patients at the time of hospital discharge, who were then referred to the cardiovascular prevention and rehabilitation service. When the patients arrived at the CVPR service, their partners were also invited to participate in the programme and both were asked to participate in an initial assessment. Patients and partners attended a weekly programme meeting over an eight-week period. The programme included group health promotion workshops and supervised exercise sessions. In each weekly session, the interdisciplinary nurse-led team, comprising nurses, a dietitian, and a physiotherapist, evaluated progress individually for all participating patients and partners, and reviewed goals for lifestyle change and risk factor management. Nurses co-ordinated the CVPR programme and provided support to all other members of the multidisciplinary team and monitored blood pressure weekly with a 711 Omron blood pressure monitor. Dietary and physical activity advice was individually tailored to help patients to maintain or improve their risk factors. The aim of the dietary intervention was to give individually tailored professional advice on food and food choices, to establish a diet associated with the lowest risk of CHD. The physiotherapist helped patients and families to increase their physical activity safely, as well as led supervised exercise sessions. On completion of the programme, the patients and partners were reassessed in the hospital at 16 weeks and one year, for lifestyle, risk factors, and therapeutic management.

Before attending the initial assessment, patients were given a number of self-administered questionnaires to complete, in Spanish, which were designed to assess their beliefs about health, their health-related quality of life (HRQoL) with the European Quality of Life-5 Dimensions (EQ-5D) and European Quality Visual Analogue Scale (EQ-VAS) [26,27,28,29], illness perception with the revised Illness Perception Questionnaire (IPQ-R) [30,31], anxiety and depression levels with the Hospital Anxiety and Depression Scale (HADS) [32,33], and positive aspects of mood with the Global Mood Scale (GMS) [34,35]. Validated versions of the questionnaires for the Spanish population were administered. Each of these questionnaires were provided once more to patients and partners at 16 weeks, on completion of the programme, and at the one-year follow-up in the intervention and usual care group. At baseline, the responder was not influenced by the programme. For that reason, the pack was administered prior to the first assessment or at least early during the first assessment. From a clinical perspective, the questionnaires were useful in obtaining an overview of the emotional and psychological state of the patient and their family, as well as an assessment of their beliefs about risk and CHD, as well as their general perceptions about the seriousness and impact they felt that the diagnosis of CHD would have on their lives.

Self-Administered Questionnaires

The HADS [36] was selected to assess psychological distress. The questionnaire included 14 items which were balanced equally between questions on depression and anxiety, although these items were mixed randomly within the questionnaire. The questionnaire is easily self-administered and contains some items that are reverse scored in order to avoid the possibility of a response set where respondents may be tempted to answer a series of questions in a certain direction regardless of their content. The questionnaire generates two summary scores, one for anxiety and one for depression. There are seven items for each dimension with a possible highest score of 21 for each. The lowest possible score for each dimension is 0. A score below 7 for each dimension is considered to be normal. A score between 7 and 10 indicates moderate levels of anxiety or depression. A score of 11 indicates severe symptoms of anxiety and depression which may warrant further investigation, although these should be seen in context. It is important to note that the HADS is not a diagnostic tool for depression.

The GMS has 10 negative and 10 positive affect items designed to measure subjective moods and feelings in the recent past, rather than investigate emotional reactions to specific events. The GMS was developed in response to the need for a disease-specific measurement of emotional distress in patients with coronary disease. In the EUROACTION study, only the positive affect items of the scale were used in order to reduce the burden of too many self-administered questions and to encourage full completion of the questionnaire. Negative emotions were assessed using a different tool, which is described below. For each descriptor, on a 5-point Likert scale ranging from “not at all” to “extremely”, patients and partners were asked to indicate the extent to which they had felt this way “lately”. The maximum score possible was 40, which would have indicated a top score in all items. The lowest possible score was 0.

The EQ-5D and EQ-VAS utility measure, developed by the Euroqol Group [26,37], provided an overall perception of health status, with a visual analogue scale, and also provided a summary of five different dimensions of HRQoL, which assesses the respondents’ perception of their health-related quality of life according to five dimensions: mobility, self-care, usual activities, pain and discomfort, and anxiety and depression. The respondents are asked to choose from three options, for which they can receive scores of 1, 2, or 3. The EQ-5D self-classifier identifies the level of problems (if any) on each of the five dimensions, and generates a weighted index. The EQ-VAS is a visual analogue scale which assesses the respondents’ perception of their state of health at any given moment, for which 0 is the worst imaginable state and 100 is the best. The patients’ rating of their health status, using the EQ-VAS at different time points, can show changes in perception of their health status over time.

**Figure 1:**
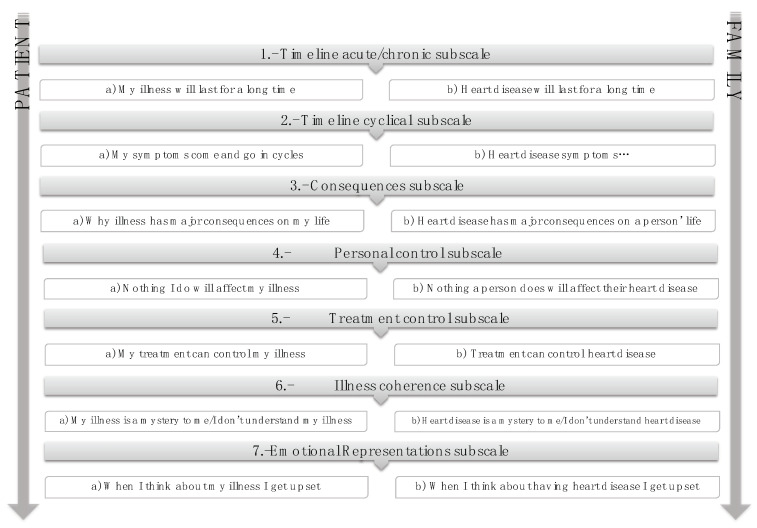
Items selected from each Illness Perception Questionnaire-Revised (IPQ-R) [30] subscale for use in the EUROACTION study.

The initial IPQ-R [38] was derived from Levanthal’s self-regulatory model of chronic illness [39] and the revised version [30] was generated to improve ratings. In the EUROACTION study [40], some items were selected from each IPQ-R subscale for use in the assessment of patients and partners (Figure 1). The questionnaire is based on the seven subscales to measure perceptions. The items are rated on a 5-point Likert scale. The questionnaire includes some items that are reverse scored. The illness perception total score is calculated as the sum of the seven individual items, giving a possible total score of 35. A higher score corresponds to a less threatening perception of the illness.

### 2.1. Measurement of Biochemical Markers

Central laboratory analysis of total cholesterol, low-density lipoprotein-cholesterol (LDL), high-density lipoprotein (HDL) cholesterol, triglycerides, and glucose was undertaken at baseline, 16 weeks, and one year. Serum concentrations of cholesterol, HDL cholesterol, and triglycerides were measured by enzymatic colourimetric tests with Roche liquid reagent assays (Roche Diagnostics, Basel, Switzerland) on a Roche 917 analyser (Roche Diagnostics).

### 2.2. Sociodemographic and Clinical Variables

The variables included sociodemographic characteristics: age, civil status, cohabitation status, educational level, and cardiovascular diagnosis.

### 2.3. Statistical Analysis

Values for continuous variables are expressed as median and interquartile range, otherwise specified by mean ± standard deviation (SD), and categorical variables as the absolute value with their percentage. The normal distribution of each variable was assessed with the Kolmorov–Smirnov test in order to determine whether a parametric or nonparametric test should be applied. A chi-square test or Fisher’s exact test was applied for categorical analyses. The correlation between quantitative variables was performed by a Spearman correlation test. Comparison between the two groups was performed by the Mann–Whitney U test or Student’s *t*-test, as appropriate, as well as by Wilcoxon’s test for paired comparison. Comparisons between the different anxiety groups were performed with the nonparametric Jonckheere–Terpstra test. Statistical significance was set at a *p*-value of less than 0.05. All statistical analyses were performed using the SPSS software package (version 26.0; SPSS, Inc., Chicago, IL, USA).

## 3. Results

### 3.1. Characteristics of the Study Population

The results are from the Spanish cohort of the global EUROACTION study. In the intervention group, 142 of the 165 patients recruited at baseline were evaluated at the one-year follow-up. In the usual care group, only a subsample of 49 patients were assessed at baseline, and 210 patients were evaluated at the one-year follow-up. The mean age was 59.87 (±10.01) in the intervention group, and 64.53 (±11.20) in the usual care group (*p* = 0.006, *t*-test). There were more men in both the intervention group (126 (76.4%)) and the usual care group (36 (73.5%)). No differences were observed in sex distribution (*p* = 0.678, chi-square test). As shown in Table 1, almost all participants were married (n = 174; 81.3%) and only 11.2% lived alone. In relation to partners, 134 (44.8%) were identified in the intervention group and 20 (30%) in the usual care group.

### 3.2. Evaluation of Psycho-Social Factors in the Study Sample

Of the whole sample, in the intervention group, 125 (75.8%) patients at baseline and 118 (71.5%) at follow-up completed the self-administered questionnaires. In the usual care group, almost all patients at baseline (49 (100%)) and at follow-up (206 (98%)), completed the questionnaires.

As shown in Table 2, when each of the four tools used to measure psycho-social factors between the intervention and usual care groups at baseline and follow-up were analysed, there were significant differences between HADS-A and HADS-D at both time points. However, anxiety levels increased at one year in the usual care group. Differences between baseline and one-year follow-up in both groups were analysed. There were significant differences in anxiety levels (*p* = 0.010, Wilcoxon test), EQ-5D (*p* = 0.008, Wilcoxon test), EQ-VAS (*p* = 0.039, Wilcoxon test), and IPQ-R score (*p* < 0.009, *t*-test) in the intervention group and in the GMS score (*p* = 0.013, Wilcoxon test) in the usual care group.

**Figure 2:**
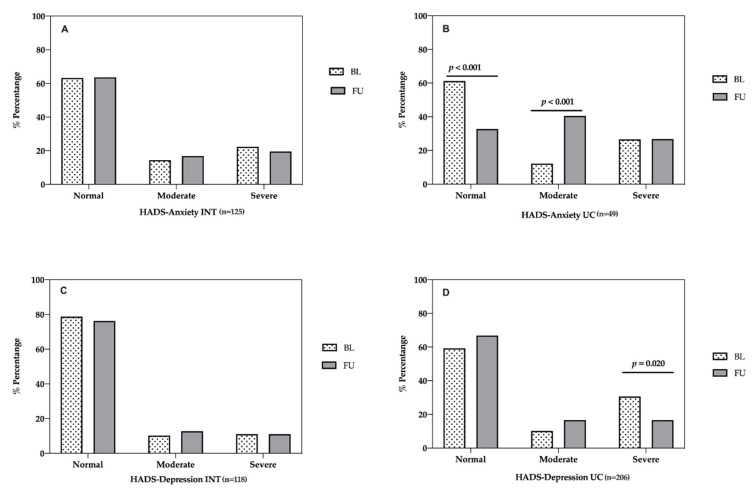
Evaluation of anxiety and depression in patients between baseline and one-year follow-up. Anxiety and depression were measured with the HADS [36]. (**A**) HADS-Anxiety levels in intervention group. (**B**) HADS-Anxiety levels in usual care group. (**C**) HADS-Depression levels in intervention group. (**D**) HADS-Depression levels in usual care group. INT = intervention. UC = usual care. BL = baseline. FU = follow-up.

In the intervention group, no significant differences were found among normal (63.2% vs. 63.6%), moderate (14.4% vs. 16.9%), and severe anxiety levels (22.4% vs. 19.5%) between baseline and one-year follow-up (Figure 2A). In the usual care group, anxiety levels worsened. There were significant differences in normal (61.2% vs. 32.7% *p* < 0.001, chi-square test) and moderate anxiety (12.2% vs. 40.5% *p* < 0.001, chi-square test) between baseline and one-year follow-up. No significant differences were found between severe anxiety levels (Figure 2B).

Related to depression, no significant differences were found between baseline and one-year follow-up in normal (78.7% vs. 76.3%) moderate (10.2% vs. 12.7%), and severe depression levels (11% vs. 11%) in the intervention group (Figure 2C). In the usual care group, there were significant differences in severe depression levels (30.6% vs. 16.6% *p* = 0.020, chi-square test) between baseline and one year follow-up. No significant differences were found among normal and moderate depression levels.

Illness perception in patients who fully participated in and completed the programme in the intervention group was analysed. There were significant differences in the personal control subscale (3.35 ± 1.17 vs. 3.48 ± 1.22, *t*-test = 0.002) and illness coherence (understanding) subscale (2.63 ± 1.31 vs. 3.30 ± 1.27, *t*-test < 0.001) between baseline and follow-up. At the one-year follow-up, there were significant difference in the illness coherence subscale (3.27 ± 1.31 vs. 2.53 ± 0.93, *t*-test < 0.001) and emotional subscale (2.75 ± 1.10 vs. 2.50 ± 0.90, *t*-test = 0.037) between patients in intervention and usual care groups.

**Figure 3:**
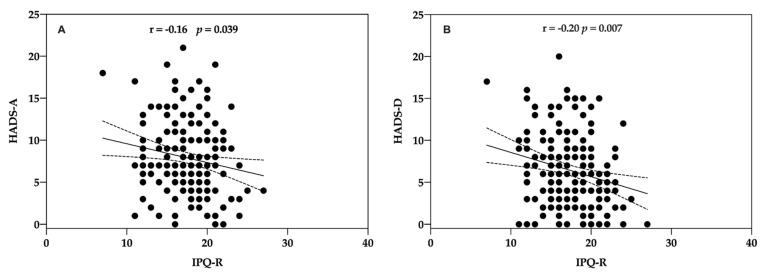
Correlation between patients’ HADS and IPQ-R total score at baseline. Illness perceptions were measured with the Illness Perceptions Questionnaire-Revised [30] and anxiety and depression with HADS [36]. (**A**) HADS-Anxiety and IPQ-R. (**B**) HADS-Depression and IPQ-R.

Illness perceptions were significantly and inversely associated with anxiety (Rho = −0.16, *p* = 0.039; Spearman test) (Figure 3A) and depression (Rho = −0.20, *p* = 0.007; Spearman test) (Figure 3B).

**Figure 4:**
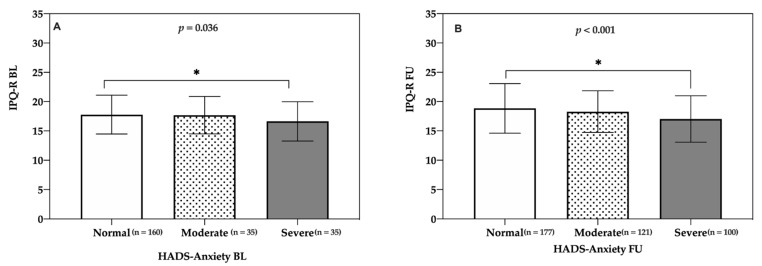
**IPQ-R.** Evaluation of the relationship between IPQ-R and HADS-Anxiety in patients and partners. (**A**) Baseline. (**B**) One-year follow-up. Illness perceptions were measured with IPQ-R [30] and anxiety with HADS [36]. Dates are expressed as the mean and standard deviation (SD) for each group. The level of significance is indicated in the corresponding panel and indicated with asterisks. BL = baseline. FU = follow-up. IPQ-R = Illness Perception Questionnaire-Revised. * *p* < 0.05.

Regarding associations between anxiety and illness perception, anxiety levels (baseline: normal (Mdn = 18; interquartile range = 5), moderate (Mdn = 18; interquartile range = 5), and severe (Mdn = 16.5; interquartile range = 5); follow-up: normal (Mdn = 19; interquartile range = 6), moderate (Mdn = 17; interquartile range = 7), and severe (Mdn = 18; interquartile range = 4)) were significantly affected by a more threatening perception of cardiovascular disease both at baseline (H(2) = 6.15) and at follow-up (H(2) = 4.86). The Jonckheere–Terpstra test revealed a significant trend in the data: as better illness perception was perceived, the median anxiety levels decreased both at baseline, J = 8490.5, z = −2.098, r = −0.13 (Figure 4A), and at follow-up, J = 20326, z = −3.651, r = −0.18 (Figure 4B). All effects reported at *p* < 0.05.

### 3.3. Association between Health Beliefs, Psycho-Social Factors, and Cardiovascular Risk Factors

As shown in Table 3, there were significant differences in health beliefs both at baseline and follow-up between intervention and usual care groups. No significant differences were found in not exercising regularly.

**Figure 5:**
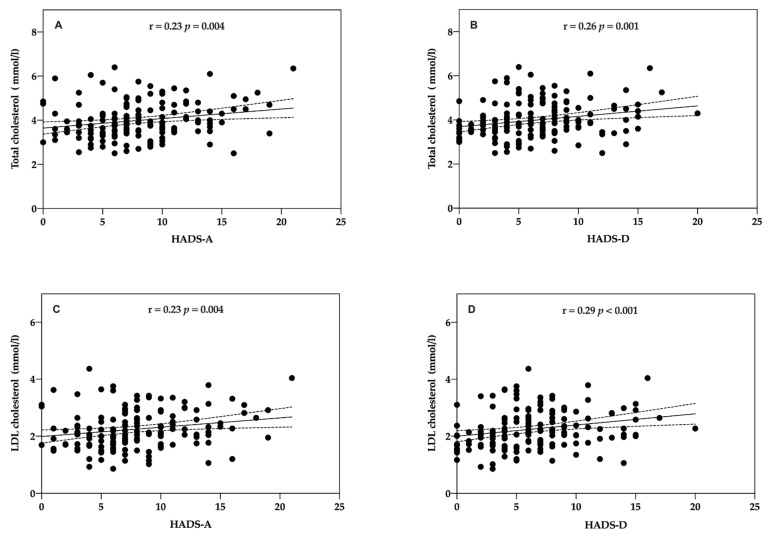
Correlation between patients’ HADS score and cholesterol at baseline. (**A**) Total cholesterol (mmol/L) and HADS-Anxiety. (**B**) Total cholesterol (mmol/L) and HADS-Depression (**C**) Low-density lipoprotein (LDL)-cholesterol and HADS-Anxiety. (**D**) LDL-cholesterol and HADS-Depression.

Anxiety and depression were evaluated with regard to whether there was correlation with cardiovascular risk factors in patients in both groups. There was a significant correlation both between anxiety and total cholesterol (Rho = 0.23, *p* = 0.004; Spearman test) (Figure 5A) and depression (Rho = 0.26, *p* = 0.001; Spearman test) (Figure 5B) and anxiety and LDL-cholesterol (Rho = 0.23, *p* = 0.004; Spearman test) (Figure 5C) and depression (Rho = 0.29, *p* < 0.001; Spearman test) (Figure 5D).

Related to patients’ lifestyle habits at follow-up, there were significant differences in fruit consumption (297.07 gr ± 123.68 vs. 192.24 gr ± 170.42, *p* < 0.001), vegetable consumption (392.25 gr ± 96.62 vs. 253.04 gr ± 86.18, *p* < 0.001, *t*-test), and systolic blood pressure (108.96 mmHg ± 47.70 vs. 140.11 ± 21.72, *p* < 0.001, *t*-test) between intervention and usual care.

### 3.4. Association of Psycho-Social Factors between Partners and Patients with Coronary Disease

**Figure 6:**
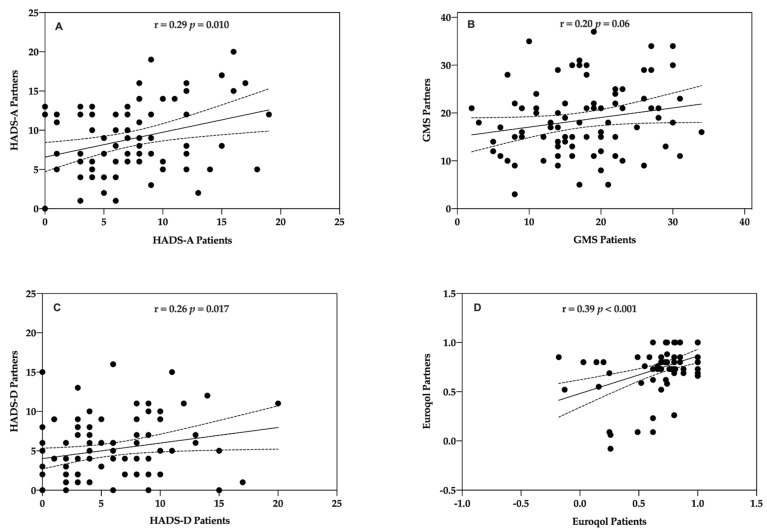
Correlation between patients and partners psycho-social factors. (**A**) HADS-Anxiety. (**B**) GMS = Global Mood Scale (0–40) [34]. (**C**) HADS-Depression. (**D**) EQ-5D [26,41].

The concordance between patients and partners for psycho-social factors at baseline was analysed by Spearman’s correlation coefficient to look for concordance between patients and partners in psycho-social factors at baseline. All of the correlations for these measures were positive and significant except for the GMS score which showed a statistically insignificant positive trend. Significant correlations were found for HADS-A (Rho = 0.29, *p* = 0.010; Spearman test), HADS-D (Rho = 0.26, *p* = 0.017; Spearman test), and EQ-5D (Rho = 0.39, *p* < 0.001; Spearman test) (Figure 6A,C,D). No significant correlation was found between patients and partners for positive mood (GMS) (Rho = 0.20, *p* = 0.056; Pearson test) (Figure 6B).

**Figure 7:**
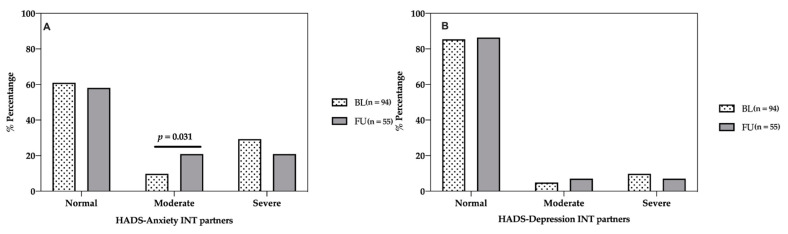
Evaluation of anxiety and depression in intervention partners between baseline and one-year follow-up. Anxiety and depression were measured with HADS [36]. (**A**) HADS-Anxiety levels. (**B**) HADS-Depression levels. INT = intervention. FU = follow-up. BL = baseline.

Related to anxiety and depression, the evolution between baseline and one-year assessment in partners from the intervention group was analysed (Figure 7). There were significant differences in moderate anxiety levels (9.8% vs. 20.9%, *p* < 0.031, chi-square test). No significant differences were found in normal and moderate anxiety or in depression levels.

There was concordance for change in lifestyle habits at one year in partners who participated in the programme. There were significant differences in fruit consumption (230.93 gr ± 127.70 vs. 269.06 gr ± 107.97, *p* < 0.001, *t*-test) and vegetable consumption (308.76 gr ± 101.68 vs. 392.30 gr ± 82.07, *p* < 0.001, *t*-test) consumption between baseline and follow-up.

## 4. Discussion

This study, which was focused on psycho-social factors of the EUROACTION family-centred CVPR, shows that patients’ and partners’ perceptions of illness were improved due to the intervention, as well as a decrease in anxiety, depression, and other cardiovascular risk factors. Moreover, the EUROACTION care model has demonstrated that family intervention is appropriate, as family members showed concordance for lifestyle, and changes in their lifestyle habits over the course of the programme.

The aim of CVPR is to promote secondary prevention and improve health-related quality of life. In order to measure psycho-social functioning in the EUROACTION study, a variety of validated patient-reported outcome measures were selected. They allowed the assessment, in both patients and partners, of their understanding and their beliefs regarding coronary disease and cardiovascular risks, and positive and negative emotional states. These outcomes are important in the context of the evaluation of CVPR [42]. The results of this study show that the programme controlled the patients’ state of anxiety and depression over time, thus improving their quality of life.

Anxiety and depression have been reliably linked with both the development of CHD and poorer prognosis post-onset [6]. For this reason, depression and anxiety levels should be assessed before and after a CVPR programme. The present study has explicitly investigated anxiety and depression levels in cardiac patients and their partners. The long-term impact of the EUROACTION intervention has shown improvements in anxiety compared to usual care. At the start of the CVPR programme (38.8% vs. 38.7%), in the intervention and usual care groups, patients had moderate to severe anxiety. Anxiety levels at follow-up improved in the intervention group after the CVPR programme (36.4% vs. 67.3%).

Among cardiac patients, anxiety is associated with a lower likelihood of adhering to a number of risk-reducing recommendations after myocardial infarction (MI), including smoking cessation, social support utilisation, and stress reduction [42,43]. Patients with anxiety disorders are also less likely to both attend and complete CVPR programmes [43].

On the other hand, by being shown cognitive representations of the disease, such as, for example, a new diagnosis of coronary disease, patients developed an organised pattern of beliefs about their condition, leading them to understand threats to their health. These explanations go on to determine their coping mechanisms [21]. The commonsense self-regulation model of health behaviour is a dynamic framework for understanding illness self-management that describes the processes involved in identifying illnesses, and in initiating and maintaining a self-regulation process to restore a state of health or mitigate threats of disease [22,44,45,46]. Prototypes and representations of a current/future health threat can have attributes in five areas: identity, timeline, consequences, causes, and control [44].

Therefore, the perception of control is an important element of the belief system of patients with chronic disease, and influences coping and the adoption of, and adherence to, health protective behaviour [21,44,47]. The results of this study show a negative association between anxiety (r = −0.16 *p* = 0.039) and depression (r = −0.20, *p* = 0.007) and illness perception. Patients with severe anxiety and levels of depression were significantly more likely to perceive CHD as more threatening. There was an association between illness representation and distress in patients with CHD. Several studies found that a strong illness identity, acute/chronic timeline, cyclical timeline, consequences, and emotional responses in stroke patients were significantly and positively related to anxiety and depression [22,23,48]. The results of this study correspond to those obtained by Nur et al. (2018). Patients with less threatening illness perception showed a positive correlation with better cardiovascular health behaviour at a significance level of 0.01 (r = 0.38, *p* < 0.01) [49]. Patients who perceived greater illness coherence and an understanding of their illness reported lower depression and lower overall distress [30].

Several pathophysiological pathways are involved in CVD pathogenic factors. Stress-induced neurotransmitter and biochemical abnormalities accompanying anxiety and/or depression would sustain a subclinical chronic inflammatory state [50,51,52]. Moreover, anxiety and depression are associated with diminished autonomic control of the heart, which may induce a higher blood pressure variability, with effects on the coronary endothelium and plaque formation [53,54,55]. Hypothalamic–pituitary–adrenal (HPA) axis dysregulation, resulting in the release of cortisol into the blood, has been identified as an unfavorable pathophysiological disturbance [51]. Kollia et al. (2017), in the ATTICA study, prospectively explored the effects of depression and anxiety on the 10-year CVD incidence [56]. They identified that psychological distress was positively associated with 10-year CVD incidence (adjusted OR per 10 units: 1.4, 95% CI: 1.1, 1.7). Three linking pathways were revealed: sedentary behaviour, inflammation, and metabolic syndrome. Furthermore, mechanisms of cardiopathogenesis, such as unhealthy lifestyles habits such as smoking, excessive alcohol use, physical inactivity, unhealthy diet, and therapy non-compliance, were attributable to anxiety and depression [51,57]. According to previous arguments, our results showed that low levels of anxiety (Rho = 0.23, *p* = 0.004) and depression (Rho = 0.26, *p* = 0.001) were associated with better self-management of total cholesterol and LDL-cholesterol (anxiety: Rho = 0.23, *p* = 0.004; depression: Rho = 0.29, *p* < 0.001). There were also significant differences in fruit consumption (297.07 gr ± 123.68 vs. 192.24 gr ± 170.42, *p* < 0.001), vegetable consumption (392.25 gr ± 96.62 vs. 253.04 gr ± 86.18, *p* < 0.001), and systolic blood pressure (108.96 mmHg ± 47.70 vs. 140.11 ± 21.72, *p* < 0.001), between intervention and usual care.

Another psycho-social factor that is important to measure in patients with coronary disease is emotional distress. Several studies have analysed the correlations between optimism and healthy behaviours [58,59]. Emotional distress in coronary patients is associated with poor recovery, worsened prognosis, and increased mortality, and a positive attitude is closely related to improved quality of life [35,60]. In our results, no significant correlation was found between patients and partners with respect to positive mood; however, the GMS score showed a statistically insignificant positive trend (Rho = 0.21, *p* = 0.056). Regarding the results of EQ-5D and EQ-VAS, patients in the intervention group reported more concern about their illness than usual care at the one-year follow-up (EQ-5D: Mdn = 0.84 vs. 0.76; EQ-VAS: Mdn = 70 vs. 60).

The EUROACTION programme promoted adoption and maintenance of positive health behaviours and self-management, by educating patients and their families on their coronary disease, its management, and treatment adherence. Chronic conditions are completely or partially asymptomatic. Treatment adherence and lifestyle changes for management require comprehensive interventions for conduct, correcting misconceptions and promoting feelings of control, resulting in the development of a less threatening view of illness [44]. In these results, patients who fully participated and completed the programme showed better personal control and understanding about cardiovascular disease. Enhanced control is related to receiving information, the ability to make decisions independently and to transform these decisions into action, and having the feeling that the outcomes of decisions are under one’s control [61,62]. As Moore et al. indicated in 2016, health professionals, particularly CVPR nurses, may facilitate more successful adaptation following CHD treatment by targeting both the psychological and knowledge-based needs of individual patients [63]. One of the main characteristics of EUROACTION is that it is a nurse-coordinated CVPR programme.

A CVPR programme, with its multifactorial approach, seems to be a good option in identifying emotional symptoms of anxiety and depression, as well as a crucial step to addressing both the psychological and educational needs of individual patients and their partners with the goal of improving their understanding of the illness [53,63]. EUROACTION was a family-centred programme, and actively recruited patients’ partners into the programme. In the longitudinal study, both patients and partners had a comprehensive lifestyle assessment, and partners participated in the programme in the same way as the patients. Family members showed concordance in anxiety, depression, and global mood score. Moreover, concordance for change was observed in more healthy eating choices. Partners and families living in the same household show concordance in lifestyle and change. A randomised community-based lifestyle intervention in patients with coronary artery disease and their partners has reported similar findings [64]. Partner participation in a lifestyle programme (137 of 298; 46%) was associated with a significantly greater success rate. Partners’ illness perceptions were important influences and would guide the patients’ habits.

The results in this study support the need to assess patients’ beliefs and illness perceptions at the start of a CVPR to promote healthy behaviours and lifestyle choices. Involving partners or caregivers in CVPR provides an important source of support for patients’ behavioural changes.

The present study has some limitations. First, the number of patients and partners recruited was smaller than expected. Second, although pairs of centres were matched, initial patient assessment revealed some unexpected differences in patient characteristics, as shown in depression levels. Third, a random subsample of usual care patients could have caused a possible underestimation of the treatment effect. Finally, as the questionnaires were self-administrated, they relied on recollection and therefore may have had a reduced reliability in responses.

## 5. Conclusions

The EUROACTION model has shown that family members build new habits together and motivate and support one another in engaging in behaviours, and continuing with them in the long-term. The EUROACTION study emphasised the importance of assessing psycho-social factors in CVPR programmes. Less threatening illness perceptions are related to improved cardiovascular health behaviours and lower anxiety and depression levels.

## Figures and Tables

**Table 1:** Sociodemographic characteristics.

Category	Variation	Intervention		Usual Care		*p*
		n	%	n	%	
Civil status	Married	136	82.4	38	77.6	<0.001
	Widower	15	9.1	8	16.3	
	Divorced	5	3.0	1	2	
	Other	9	5.5	2	4.1	
Cohabitation status	Single	16	9.7	8	16.3	0.592
	With partner/family	149	90.3	41	83.7	
Educational level	Without studies	7	4.2	2	4.1	0.002
	Primary	84	50.9	20	40.8	
	Secondary	29	17.6	22	44.9	
	University	14	8.5	1	2	
Professional situation before CV event	Full-time worker	76	46.1	8	16.3	<0.001
	Part-time worker	2	1.2	1	2	
	Self-employed	10	6.1	2	4.1	
	Unemployed	6	3.6	1	2	
	Retired	58	35.2	25	51	
	Other	13	7.8	12	24.5	
CV diagnosis	AMI	122	75.8	23	48.9	<0.001
	Unstable angina	31	19.3	23	48.9	
	Stable angina	8	5	1	2.1	

CV = cardiovascular, AMI = acute myocardial infarction.

**Table 2:** Patients psycho-social factors at baseline and follow-up.

Psycho-Social Characteristics	Baseline					Follow-Up				
	INT (n = 125)		UC (n = 49)		*p*	INT (n = 118)		UC (n = 206)		*p*
	Median/ Mean Value	IQR/ ±SD	Median/ Mean Value	IQR/ ±SD		Median/ Mean Value	IQR/ ±SD	Median/ Mean Value	IQR/ ±SD	
HADS-Anxiety	7.0	6.0	8.0	5.0	0.024	6.0	5.0	9.0	4.0	<0.001
HADS-Depression	5.0	6.0	8.0	5.0	<0.001	5.0	6.25	7.0	4.0	<0.001
GMS	17.21	7.77	19.0	9.50	0.836	18.17	6.72	21.5	7.5	<0.001
EQ-5D	0.79	0.29	0.72	0.16	0.044	0.84	0.31	0.76	0.19	0.048
EQ-VAS	70.0	30.0	60.0	25.0	0.219	70.0	23.50	60.0	10.0	0.001
IPQ-R	17.38	3.46	17.75	2.57	0.430	18.35	3.77	18.0	5.00	0.726

HADS = Hospital Anxiety and Depression Scale (HADS) [36], GMS = Global Mood Scale (0–40) [34], EQ-5D = European Quality of Life-5 Dimensions [37], EQ-VAS = European Quality Visual Analogue Scale [37], IPQ-R = Illness Perception Questionnaire-Revised [30]. INT = intervention. UC = usual care.

**Table 3:** Health beliefs at baseline and follow-up.

	Baseline					Follow-Up				
	INT (n = 142)		UC (n = 49)		*p*	INT (n = 119)		UC (n = 206)		*p*
Does the Patient Believe that the Following Behaviours are Harmful to His/Her Health	Median	IQR	Median	IQR		Median	IQR	Median	IQR	
**Smoking**	4.0	2.0	2.0	1.0	<0.001	4.0	1.00	3.0	2.0	<0.001
**Being overweight (10 kgs or more)**	3.0	1.0	2.0	1.0	<0.001	3.0	1.00	3.0	1.0	<0.001
**High-fat diet**	3.0	1.0	2.0	1.0	<0.001	3.0	1.0	3.0	1.0	<0.001
**Drinking heavily**	3.0	1.0	3.0	1.0	0.001	4.0	1.0	3.0	2.0	<0.001
**Not exercising regularly (at least 3 to 4 times a week)**	2.0	2.0	2.0	1.75	0.220	3.0	1.0	3.0	1.0	0.289
**Feeling under regular stress (at least twice a week)**	3.0	2.0	2.0	2.0	0.010	3.0	1.0	3.0	1.0	<0.001
**Not taking prescribed medication**	4.0	2.0	2.0	1.0	<0.001	4.0	2.0	3.0	2.0	<0.001

1.0 = not harmful; 2.0 = somewhat harmful; 3.0 = quite harmful; 4.0 = very harmful; 5.0 = extremely harmful.

## Data Availability

The data presented in this study are available on request from the corresponding author. The data are not publicly available due to [their containing information that could compromise the privacy of research participants].
